# A conceptual framework for the dynamic modeling of time-resolved phenotypes for sets of genotype-environment-management combinations: a model library

**DOI:** 10.3389/fpls.2023.1172359

**Published:** 2023-06-14

**Authors:** George A. K. van Voorn, Martin P. Boer, Sandra Huynh Truong, Nicholas A. Friedenberg, Shota Gugushvili, Ryan McCormick, Daniela Bustos Korts, Carlos D. Messina, Fred A. van Eeuwijk

**Affiliations:** ^1^ Biometris, Plant Sciences Group, Wageningen University & Research, Wageningen, Netherlands; ^2^ Research & Development, Corteva Agriscience, Johnston, IA, United States; ^3^ Gro Intelligence, New York, NY, United States; ^4^ Institute of Plant Production and Protection, Faculty of Agricultural Sciences, Universidad Austral de Chile, Valdivia, Chile; ^5^ Department of Horticultural Sciences, University of Florida, Gainesville, FL, United States

**Keywords:** crop growth model, ordinary differential equation, dynamic model, genotype by environment by management interactions, model selection, phenomenological model, phenotyping, process-based model

## Abstract

**Introduction:**

Dynamic crop growth models are an important tool to predict complex traits, like crop yield, for modern and future genotypes in their current and evolving environments, as those occurring under climate change. Phenotypic traits are the result of interactions between genetic, environmental, and management factors, and dynamic models are designed to generate the interactions producing phenotypic changes over the growing season. Crop phenotype data are becoming increasingly available at various levels of granularity, both spatially (landscape) and temporally (longitudinal, time-series) from proximal and remote sensing technologies.

**Methods:**

Here we propose four phenomenological process models of limited complexity based on differential equations for a coarse description of focal crop traits and environmental conditions during the growing season. Each of these models defines interactions between environmental drivers and crop growth (logistic growth, with implicit growth restriction, or explicit restriction by irradiance, temperature, or water availability) as a minimal set of constraints without resorting to strongly mechanistic interpretations of the parameters. Differences between individual genotypes are conceptualized as differences in crop growth parameter values.

**Results:**

We demonstrate the utility of such low-complexity models with few parameters by fitting them to longitudinal datasets from the simulation platform APSIM-Wheat involving *in silico* biomass development of 199 genotypes and data of environmental variables over the course of the growing season at four Australian locations over 31 years. While each of the four models fits well to particular combinations of genotype and trial, none of them provides the best fit across the full set of genotypes by trials because different environmental drivers will limit crop growth in different trials and genotypes in any specific trial will not necessarily experience the same environmental limitation.

**Discussion:**

A combination of low-complexity phenomenological models covering a small set of major limiting environmental factors may be a useful forecasting tool for crop growth under genotypic and environmental variation.

## 1 Introduction

The prediction of crop yield remains a critical challenge (Peng et al., 2020). Yield is a complex trait, resulting from the interplay between many genes with relatively small contributions, environmental inputs, and management regimes (GxExM) integrated over the growing season (Van Eeuwijk et al., 2019). Crudely speaking, two major methodological approaches can be distinguished to decompose yield into genetic and environmental factors. The first approach is mainly statistical, where the underlying goal is yield improvement by the identification of superior genotypes through the use of relatively simple models in which genotypes differ from each other in mean performance across the range of included conditions and in sensitivities to either a small (Millet et al., 2019) or large number of environmental drivers (Jarquín et al., 2014; Van Eeuwijk et al., 2016). The second approach involves the use of process-based models, which typically relate yield to underlying processes that are affected by the environment and that are governed by genotype-dependent parameters that are expected to vary little across environments. The use of such models is more common in integrated assessments, such as implemented in AgMIP (Ruane et al., 2017), where the goal is to assess the effects of management and stress factors on yield, such as those resulting from climate change. Yield can also be disentangled following a prior dissection of yield into ‘lower-level’ yield components that are physiologically simpler than yield and that can be measured at multiple moments during the growing season, for example, with High Throughput Phenotyping platforms. Static yield dissection models may be applied that can better address equifinality issues, i.e., the same yield end point is achieved via different development paths, by allowing for the possible improvement of yield along multiple paths via different underlying components (Tsutsumi-Morita et al., 2021). Longitudinal data series of yield components allow dynamic modelling in the form of smoothed growth curves on P-spline bases in combination with extraction of growth characteristics that can be used to predict end point traits like yield (Roth et al., 2021; Pérez-Valencia et al., 2022). The disadvantage of such models is that they do not explicitly couple mechanistic descriptions of the underlying dynamic processes driving crop growth to genetic effects. Vice versa, crop simulation platforms integrate environmental factors over the growing season to forecast yield as an emergent end point. While mechanistic in nature, these models do not usually involve genetic differences, and the inclusion of such effects is far from trivial, as it is not obvious which crop growth parameters to choose to be genotype dependent and how to account for stochasticity in genetic effects. Moreover, despite the increasing availability of spatio-temporal information from non-destructive, cost-effective, and time-efficient methods (Shammi & Meng, 2021), such as longitudinal drone imagery (Panday et al., 2020) and earth observations freely available at relevant scales (Kasampalis et al., 2018; Huang et al., 2019), considerable limitations exist with respect to the availability of data, models, and algorithms to adequately handle GxExM in crop growth descriptions (Stöckle & Kemanian, 2020).

The generalization of crop growth models to contain genotype-dependent parameters is relevant for increasing the accuracy of predictions regarding the performance of genotypes in new environments (Technow et al., 2015). With thousands of genotype-by-environment combinations involved in modern breeding, there is a need for crop models that can cover the broad spectrum of GxExM interactions and make optimal use of the data that are becoming available. On the other hand, these models should also be sufficiently simple and parsimonious to aid human interpretation (Hammer et al., 2019). While many current crop simulation platforms are physically consistent (e.g., containing conservation of mass and energy) and are capable of simulating crop growth and development in great detail, crop model results are sensitive to calibration, i.e. estimation of crop growth parameters in the light of empirical data is cumbersome (Grassini et al., 2015). The number of parameters in a model can quickly outpace the ability to fully identify and/or estimate all parameters well from available data when considering a single calibration objective only (Wagener et al., 2003). Crop models currently may be too complex for proper calibration so that many uncertainties remain regarding their parameterization (Dokoohaki et al., 2021). Parameters are commonly correlated in such a way that their effects on the model output are indistinguishable, leading to what is termed unidentifiability (Cole et al., 2010). Modelling efforts also suffer from the existence of multiple candidate model parameterizations and model structures that can describe or explain the data equally well (Beven & Freer, 2001), yet suggest contradictory assessments when focused on practical problems such as yield gap estimates (Schils et al., 2022). High parameter correlations and equifinality are issues that can easily disrupt attempts at an accurate estimation of parameters and thus should be addressed to avoid a reduction in the utility of crop models (Lamsal et al., 2018). Given the right tools, the availability of high-resolution time series data can help in addressing these issues.

The burden of complexity in data-driven modeling calls for the periodic reassessment of simpler approaches to identify necessary and sufficient levels of detail, or granularity, to capture the essential GxExM interactions and utilize increasingly available data streams, while also being sufficiently realistic in the sense of trying to minimize issues around model structure identification and model calibration. In this paper and a follow-up paper the over-arching aim is the development of a modelling framework for describing the essential dynamical growth patterns of genotypes that lead to GxExM interactions, which should allow for the prediction of yield for existing and new genotypes across a wide range of management and environmental conditions, i.e. it is minimalistic yet sufficiently capable of allowing for genotype-dependent parameterizations. Model complexity should be balanced in terms of what is required by the application, the important characteristics of the system – those addressing essential GxExM interactions – and the support following from data (Wagener et al., 2001).

We present a small library consisting of four crop growth models based on differential equations for the dynamical description of biomass growth during the growing season and the interaction of biomass with important environmental drivers. For simplicity, we assume that biomass is proportional to the whole crop biomass, though only above-ground biomass is measured. Furthermore we assume that the end-of-season biomass is an approximation to yield. The proposed models each focus on one particular crop growth limitation. They omit phenological stages and thereby avoid the need for stage-specific parameterization. Differential equations typically have no closed-form solutions, which prohibits the use of regular statistical methods for data fitting or model analysis. In the current paper, we demonstrate the utility of the different models from the library by fitting them to longitudinal data of individual genotypes in individual environments, using established fitting procedures for ordinary differential equations. In a follow-up paper we will develop a hierarchical Bayesian framework to fit the models within our library to longitudinal data for populations or panels of genotypes with the hope of identifying genotype-dependent parameters that do show variation across genotypes while varying little across environments. Various options exist for combining the different models in our library to arrive at a prediction model for yield, an attractive one consisting in an ensemble model (Hoeting et al., 1999; McCormick et al., 2021) that encloses the fits of the member models of the library for the time series data of individual genotypes.

In this paper, we will fit our models to simulated, i.e., *in silico* generated and noise-free longitudinal data of daily biomass measurements for different wheat genotypes in different Australian environments. The advantage of using simulated data is that we have practically unlimited data available for model testing, and we can – for now – ignore uncertainty resulting from noise and poor temporal coverage. Genetic effects will occur as differences in the estimated values for the parameters. We acknowledge that parameters that show genetic variation in our model fits are not necessarily immediately useful for prediction of yield under all conditions. It is obvious that we will need to verify that parameters with genetic variation are not subject to genotype-by-environment interaction themselves (Lamsal et al., 2018). Hammer et al. (2006) state that fundamental physiological parameters should have fixed values. For our low-complexity models we do not necessarily expect that parameters are stable, because the modelled processes are high-level, and parameter values may be the net result of multiple underlying processes. Still, we believe that even when our dynamic parameters show some sensitivity to the environmental conditions, our models can be useful for yield prediction as long as this sensitivity can be modelled itself as a simple function of the environmental conditions.

## 2 Materials and methods

### 2.1 Proposed phenomenological models

Below we introduce four dynamic models, all containing some essential first principles of crop growth. The models we develop are extensions of general continuous model frameworks presented in the literature suitable for describing the growth of plants in an ecological context (Paine et al., 2012) or the within-season accumulation of crop biomass (Poudel et al., 2022). Though simple and largely phenomenological, the models are dynamic and therefore offer a biological interpretation of the parameters as well as the ability to produce varied output depending on environmental or management inputs. Variations in genotypic background can be conceptualized as differences in the values of these parameters, where we do not exclude that the values of these parameters may still be subject to some genotype by environment interaction, especially in situations where multiple limiting factors influence our phenotype biomass. The models we present are smooth in that they lack pre-determined non-linearities, such as imposed jumps, switches, and thresholds, that are often encountered in crop simulation platforms that contain connected sub-modules for different crop processes. As such, differential equation-based models can capture the dynamic nature of crop growth in explicit descriptions. An additional advantage of this smoothness is the access to higher-order derivatives with respect to time that can be used to calculate genotype-dependent sensitivities of growth to environmental inputs as function of time as well as the timing of critical developmental events. This also conceptually facilitates the extrapolation to other genotypes and environments, assuming that the base model is valid. In addition, smooth models are easier to fit.

#### 2.1.1 Model #1: The logistic model

Model #1 – commonly referred to as the logistic model – is often used for the coupling of growth rate to biomass (Richards, 1959). For example, this model was already used more than a century ago to describe sunflower (*Helianthus*) growth (Reed and Holland, 1919). The model description is as follows:


Eq. (1) $$\frac{dM(t)}{dt}=r\;M(t)\;\left(1-\frac{M(t)}{M_{max}}\right)$$

With explicit solution:


$$M(t)=\frac{M_{max}}{1+\left(\frac{M_{max}}{M_{0}}-1\right)\text{exp(-}rt)}$$

The model symbols are given in **Table 1**. Here *M*(*t*) indicates the total biomass at time *t*. The parameter *r* is an intrinsic growth rate (with a positive value), and *M_max_* is an intrinsic (implicit) growth limitation (also with a positive value), i.e., the crop cannot grow larger than a maximum size. The initial condition *M*_0_ (with a positive value) can be interpreted as seed weight or the biomass at the starting day of measurement (depending on the context); in the data we use, it is the biomass at the first day at which biomass can clearly be observed. Genotypic variation can be represented as variation in the parameters *r*, *M_max_*, and possibly the initial condition *M*_0_ – in which case it would represent genotypic differences in seed size and reserve content, and hence all three parameters/initial conditions are candidates for genotype-dependent parameters that require estimation for each included genotype. The logistic model has an explicit solution (given in Eq. 1) that can be used to verify the numerical implementation of the model. An important ramification of this property is that the biomass development for the growing season is fixed by the initial biomass *M*_0_ together with the parameter values, in other words, the explicit solution means that no modifications of the growth rate during the growing season are accounted for in the forecasting by the model, and thus the end-of-season biomass is ‘fixed’. This is likely unrealistic, as in reality the crop growth rate may be modified by externally imposed limitations occurring during the growing season. The logistic model cannot reproduce mid-season biomass loss that can occur, for instance, when resource acquisition falls short of maintenance respiration needs (Cannell and Thornley, 2000). Additionally, logistic growth has also been shown to violate mass balance assumptions (Kooi et al., 1998).

**Table 1:** Symbols introduced in the logistic model (Eq. 1), alphabetically ordered.

Symbol	Meaning	Units	Type of parameter
*t*	Time	day	Autonomous state variable
*M*(*t*)	Crop biomass	kg m^-2^	Non-negative state variable
*M*(0)	Initial crop biomass	kg m^-2^	Non-negative initial condition *M*(*t*)=*M*_0_ at *t*=0
*M_max_*	Natural crop biomass limitation	kg m^-2^	Non-negative parameter
*r*	Crop intrinsic growth rate	day^-1^	Non-negative parameter

Parameters and initial conditions are included in the fitting procedure, unless stated otherwise.

#### 2.1.2 Model #2: The irradiance model

Model #2 – the irradiance model – assumes sunlight is the limiting factor during the growing season. The model description is as follows:


Eq. (2) $$\frac{dM(t)}{dt}=\left(r+A\;\text{sin}\left(\frac{2\text{π}}{365}\left(t+\varphi \right)\right)\right)\;M(t)\left(1-\frac{M(t)}{M_{max}}\right)$$

The newly introduced symbols in this model are given in **Table 2**. The base intrinsic growth rate *r* is now modified by the sinusoidal driver function with amplitude *A* and phase shift *φ*. This function links the level of irradiance to the yearly earth’s orbit around the sun. This implies that the parameters *A* and *φ* may vary across environments but also may be genotype-dependent, as different genotypes may respond differently to the same environmental input. The advantage of this formulation is that no input is needed and hence no additional equations are needed for translating such input. This comes at the cost of ignoring day-to-day variations in the irradiance, e.g., resulting from clouds. Instead it assumes the generic seasonal pattern of increasing and decreasing day length and changing angle of sunlight reception. In principle one could opt for the inclusion of day-to-day irradiance measurements, in which case a data smoothing and translation function is needed similar to what we use for smoothing and translating temperature (see model #3). By selecting the correct phase shift, the growth rate can increase and decrease following seasonal effects. Parameters *A* and *φ* can both have positive and negative values, depending on location and timing; care should be taken that the total term does not become smaller than *r* to avoid negative growth, though a negative growth can occur for a small amount of time as long as biomass remains positive. In case *A*=0, the model collapses to the logistic model. The other parameters have the same meaning as in the logistic model (Eq. 1).

**Table 2:** Symbols introduced in the irradiance model (Eq. 2), alphabetically ordered.

Symbol	Meaning	Units	Type of parameter
*A*	Amplitude of time-dependent driver	day^-1^	Parameter, genotype and environment-dependent
*φ*	Phase shift of driver	day	Parameter, genotype and environment-dependent

#### 2.1.3 Model #3: The temperature model

Model #3 – the temperature model – includes an explicit effect of temperature on the growth rate. The model description is as follows:


Eq. (3a) $$\frac{dM(t)}{dt}=rf_{T}(t)M(t)\left(1-\frac{M(t)}{M_{max}}\right)$$

Eq. (3b) $$f_{T}(t)={\left(1+\text{exp}\left(\frac{T_{AL}}{T(t)}-\frac{T_{AL}}{T_{L}}\right)+\text{exp}\left(\frac{T_{AH}}{T_{H}}-\frac{T_{AH}}{T(t)}\right)\right)}^{-1}$$

The base growth rate *r* in Eq. (3a) is modified by a function *f_T_* that depends on the actual ambient temperature *T*(*t*), which is a variable given by the data. The temperature response description has been identified as a major source of uncertainty in simulation models used for crop growth predictions (Wang et al., 2017; Roth et al., 2022). The temperature response curve is often included in crop models as either a linear increase in the development rate from a given base temperature (usually zero degrees Celsius), and a linear decline in biomass growth beyond a certain maximum temperature, without assuming any optimal growth temperature, or a function that includes a minimum and an optimal temperature but without a maximum temperature, thus ignoring any effects of heat stress and senescence (Wang et al., 2017; see their Figure 1 for the different types of temperature response curves). Both response curves are unlikely, as crops have three cardinal temperatures. Wheat, for example, is sensitive to high temperatures during several developmental phases, while optimal temperatures for anthesis and grain filling are given around 12 to 22°C (Djanaguiraman et al., 2018) or up to 25°C for optimal growth in general, and with minimum and maximum growth temperatures of 3–4°C and 30–32°C, respectively (Porter and Gawith, 1999; Curtis, 2002).

The exact formulation of the temperature response is debatable. Different models with unimodal temperature dependence are reviewed by DeLong et al. (2017), who alternatively consider enzyme-assisted Arrhenius temperature responses. Arroyo et al. (2022) propose a general theory for temperature dependence based on Eyring-Evans-Polanyi’s theory for chemical reaction rates. Here, we use the temperature response curve Eq. (3b) proposed by Sharpe and DeMichele (1977) and later Kooijman (2010). This formulation is based on the concept that enzymes become inactive at temperatures that are too high or too low. The reaction rate is multiplied by the active enzyme fraction, which is assumed to be in equilibrium. This formulation gives a smooth, nonlinear function that is based on three cardinal temperatures. Its parameters are given in **Table 3**. *T_L_* and *T_H_* represent the lower and upper boundary of the tolerance range, respectively, while *T_AL_* and *T_AH_* give the Arrhenius temperatures for the rate of decrease at these respective boundaries. The parameters for temperature response in Eq. (3b) were kept fixed at *T_L_* =292K, *T_H_* =303K, *T_AL_* =20,000K, and *T_AH_* =60,000K to generate a response curve that approximates the reported cardinal temperatures for wheat (Parent and Tardieu, 2012).

**Table 3:** Symbols introduced in the temperature model (Eq. 3), alphabetically ordered.

Symbol	Meaning	Units	Type of parameter
*T*(*t*)	Ambient temperature	*K*	Spline-smoothened input from daily measurements
*T_AH_*	Arrhenius temperature for the rate of decrease at the upper boundary of the temperature tolerance range	*K*	Kept constant at 6·10^4^
*T_AL_*	Arrhenius temperature for the rate of decrease at the lower boundary of the temperature tolerance range	*K*	Kept constant at 2·10^4^
*T_H_*	Upper boundary of the temperature tolerance range	*K*	Kept constant at 303
*T_L_*	Lower boundary of the temperature tolerance range	*K*	Kept constant at 292

#### 2.1.4 Model #4: The soil water model

Model #4 – the water model – involves water limitation, taking into account that many crop-growing environments are water-limited. The model description is as follows:


Eq. (4a) $$\frac{dW(t)}{dt}=p\left(\frac{M(t)+Kq}{M(t)+K}\right)P\left(t\right)-c\left(\frac{W(t)}{W(t)+n}\right)M{\left(t\right)}^{v}-RW(t)$$

Eq. (4b) $$\frac{dM(t)}{dt}=gc\left(\frac{W(t)}{W(t)+n}\right)M{\left(t\right)}^{v}-mM(t)$$

The symbols used in this model are given in **Table 4**. Contrary to temperature and irradiance, which are exogenous inputs for crop growth, there is a feedback between soil water and the crop, as one affects the other. The water model therefore includes a second differential equation that describes a soil water variable, *W*(*t*), as well as a function that couples soil water to crop biomass growth. The water model is based on a formulation proposed for simulating plant growth in semi-arid areas (Van de Koppel and Rietkerk, 2004) with some modifications.

**Table 4:** Symbols introduced in the water model (Eq. 4), alphabetically ordered.

Symbol	Meaning	Units	Type of parameter
*c*	Soil water uptake capacity of crop (*c_max_* in KR2004)	kg^-1^ ml m^-1^ day^-1^	Non-negative genotype-dependent parameter
*g*	Soil water to crop biomass conversion factor (comparable to *g_max_* in KR2004, but note the difference to our *gc*)	kg ml^-1^ m	Non-negative genotype-dependent parameter
*K*	Infiltration constant related to crop (*k* in KR2004)	kg m^-2^	Non-negative parameter that depends on genotype and environment
*n*	Half-rate parameter of Michaelis-Menten function (*k*_1_ in KR2004)	ml m^-3^	Non-negative genotype-dependent parameter
*m*	Density-dependent maintenance rate (comparable but not similar to (*d*+*δP*) in KR2004)	day^-1^	Non-negative genotype-dependent parameter
*P*(*t*)	Precipitation (observed) (*PPT* in KR2004)	ml m^-2^ day^-1^	Spline-smoothed input from daily measurements
*p*	Conversion constant from precipitation *P*(*t*) to soil water *W*(*t*) (missing in KR2004)	m^-1^	Estimated environment-dependent parameter
*q*	Fraction of precipitation that infiltrates in soil (W_0_ in KR2004)	–	Estimated environment-dependent parameter
*R*	Soil drying rate (*r_w_* in KR2004)	day^-1^	Estimated environment-dependent parameter
*v*	Parameter relating uptake volume to biomass (missing in KR2004)	–	Genotype-dependent parameter with range 0 < *v ≤* 1
*W*(*t*)	Soil water	ml m^-3^	Non-negative state variable

For easy comparison, we also list the symbols used in the original model by Van de Koppel & Rietkerk (2004) – abbreviated as KR2004 – but note there is not a one-to-one match to the model in that paper.

The first nonlinear term in Eq. (4a) represents the uptake of precipitation by the soil, which has limited uptake capability. The uptake increases with biomass, representing an increasing infiltration because of larger root structures. However, the uptake fraction remains small and under zero biomass, this term reduces to *pqP*(*t*), representing the infiltration of precipitation in barren soil. Parameters *p* and *q* depend on environmental conditions and are expected to vary across environments but not across genotypes. Parameter *K* represents infiltration of water into the soil, which is affected by the root structure of the crop, and this parameter is therefore expected to depend on genotype and environment.

The second nonlinear term in Eq. (4a) represents the uptake of water from the soil by the crop, which is limited by aquaporin (enzyme) activity and diffusion rates and thus described by a Michaelis-Menten function, with a half-rate parameter, *n*. This function mimics satiation, as the response is near-linear for small values of *W*(*t*), but gradually approaches 1 as *W*(*t*) continues to increase. Parameter *v* represents a scaling between the uptake surface of the crop and the volume over which maintenance is paid (Kooijman, 2010, Figure 4.14), where *v* is typically smaller than 1. Parameters *c*, *n* and *v* are involved in the uptake of soil water by the crop and are hence expected to be genotype-dependent.

Conceptually, in this model the crop ‘competes’ with the soil for water, and water is taken up from the soil either by the crop, or water disappears via the third term in Eq. (4a), *RW*(*t*). This is a generic term that conceptually considers the soil to be a ‘leaky bucket’, where the drying out rate depends on soil water content. If unused, water will also disappear from the soil. Parameter *R* is therefore assumed to be environment-dependent. The *de facto* standard for evapotranspiration in crops for the EU and US is the Penman-Monteith method, covered by the FAO56 method (Pereira et al., 2015), which includes effects of temperature and wind speed on how fast water disappears from the soil. Evaporation from barren soil is the largest contributing factor for water loss from the soil early in the growth season. If biomass *M*(*t*)=0 and under constant precipitation – and implicitly assuming a constant temperature, wind speed, etc. – Eq. (4a) will eventually reach a steady state, i.e. the same amount of water will enter and leave the soil in a given time interval. If there is no precipitation, the leaky bucket formulation ensures the model will eventually approach the limit *W*(*t*)=0. In reality, the soil will never fully dry out because some water is retained through gravitational and capillary forces, but this water would also not be available to crops, so we ignore this feature. Moreover, the drying and re-wetting curves of soil moisture content as functions of water pressure differ (Shein & Mady, 2018) which is also ignored in the water model. Finally, we assume there is no measurable effect of temperature, wind speed, etc. on the rates at which water leaves the soil. Note, that in our fitting procedure we not only compare the predicted biomass *M*(*t*) to the APSIM simulated biomass, but we also compare the variable *W*(*t*) to the soil water output of the APSIM SoilWat module (Holzworth et al., 2018). This makes use of real-life environmental data like rainfall and temperature, but simulates soil water in time. Hence, this implies we have a considerable reduction in model complexity.

The first term in Eq. (4b) gives the conversion from taken up water from to soil to crop biomass, i.e. water is used in the creation of carbohydrates (photosynthesis). We consider this conversion to follow a fixed ratio, and hence the parameter *g* is also fixed. Part of the taken up water will also disappear again through evapotranspiration, but we assume this ratio now to be fixed as well. The negative density-term in the second term of Eq. (4b) represents maintenance respiration, which is considered to be proportional to biomass volume (Kooijman, 2010). This term allows for a temporary decrease in biomass (rate).

### 2.2 Data description and software implementation

To assess the suitability of our four minimalistic crop growth models in capturing biomass growth in real-life situations, we used simulated longitudinal biomass data for an Australian diversity panel with 199 genotypes in wheat (Bustos-Korts et al., 2019a; Bustos-Korts et al., 2019b). These data were generated with the crop growth simulation platform APSIM-Wheat (Keating et al., 2003; Holzworth et al., 2018). Environmental inputs were observed soil and meteorological data for 31 years (1993 through 2013) at four different locations in Australia – Emerald (–23.53 lat, 148.16 long), Merredin (–31.50 lat, 118.22 long), Narrabri (–30.32 lat, 149.78 long), and Yanco (–34.61 lat, 146.42 long) (Bustos-Korts et al., 2019b). The overall data set consisted of 23,880 output series of daily observations on simulated biomass and other traits. Genotypic specifications of crop growth parameters were chosen to mimic realistic genetic variation for Australian environmental conditions (Bustos-Korts et al., 2019a; Bustos-Korts et al., 2019b). We preferred to use simulated wheat data over data from field trials, because for our simulated data we could infer to a certain extent what the major stress had been to which the wheat genotypes were exposed in a particular simulated experiment. The latter information helped us to assess the appropriateness of our candidate minimalistic models when fitted to the simulated longitudinal biomass data for an individual genotype in a simulated experiment. We did not add noise to the APSIM simulated data because we wanted to establish the performance of our minimalistic models under the most discriminatory conditions. The simulated data covered multiple types of environments with different limitations (Bustos-Korts et al., 2019a; Bustos-Korts et al., 2019b).

Typically observations on biomass consist of measurements of the state variable biomass at specific time points, and not the rate parameters that determine biomass change. We therefore need to fit solutions of our candidate minimalistic crop growth models to the simulated wheat data to obtain estimates for the rate parameters and predictions for the state variables by numerical integration. The models were implemented in R version 4.1.0 (R Core Team, 2021). Parameter estimation methods for differential equations are discussed, for instance, by Ashyraliyev et al. (2009). For model fitting we used the R package ‘FME’ (Soetaert and Petzoldt, 2010), in combination with the R package ‘deSolve’ (Soetaert et al., 2010). This combination is tailored at fitting differential equations. The default solving option we used is ‘Marq’ (short for Levenberg-Marquardt), which is a gradient-based method that minimizes the sum of squared residuals. This method is fast, but it is known to be sensitive to the initial parameter vector, because by following the steepest descending gradient it can easily end up in a local minimum. We also used the alternative methods ‘Nelder-Mead’ and ‘Pseudo’ for crude-but-fast convergence to approximate solutions in cases where ‘Marq’ did not immediately provide satisfactory solutions. Other alternatives in the FME package include SANN (simulated annealing) and bobyqa; alternative fitting packages in R include Particle Swarm Optimization (Bendtsen, 2022) and Differential Evolution (Ardia et al., 2010; Mullen et al., 2011). As the focus in this paper is not on the parameter estimation *per se*, we selected the default option in FME, which is Marq (but note that we will focus on parameter estimation in the next paper). To enhance estimation procedures, biomass was rescaled by division by 1000.

The quality of the fits was evaluated by the inspection of the plots of the weighted residuals against time, which are defined as


Eq. (5) $$r_{i}=f\left(x_{i}\right)-y_{i}$$

Here *y_i_* is the observation at index *i*, and *f*(*x_i_*) is the model predicted value. To compare the quality of the fits between models to the APSIM data, we heuristically looked at Akaike’s Information Criterion, AIC (Akaike, 1974)


Eq. (6) $$\text{AIC}=-2\left(\text{LL}\right)+2\;\#\left(\theta \right)$$

Here LL is the log-likelihood, taken as the sum of squared errors which is given by the FME package, and #(*θ*) is the total number of parameters. Note, that the models are not nested – with the exception of model #2 that can be collapsed into model #1 under the conditions stated earlier. The calculated AICs should therefore be interpreted as an assessment of the ability of the models to capture the crude patterns in the data rather than a quantitative model selection criterion. For real dynamic data, residuals will show various forms of autocorrelation. Such autocorrelations can be inspected by plots of partial autocorrelation functions, or PACFs (Hyndman and Khandakar, 2008; Hyndman et al., 2023) and tested by Durbin-Watson statistics (Fox & Weisberg, 2019). For our APSIM wheat data we did not add independent errors, and therefore the utility of inspections of residuals on autocorrelation is limited. In our follow-up paper, these issues will be revisited in the context of a hierarchical Bayesian framework for fitting the dynamics of a collection of genotypes across a series of trials and environmental conditions.

The parameters for temperature response in Eq. (3b) were kept constant across genotypes implying there is no genetic variation for these parameters. The fitting of the temperature model involved the use of penalized splines (R package ‘pspline’; Ramsay and Ripley, 2022) to smooth and interpolate daily temperatures for input to Eq. (3b). Daily rainfall was similarly smoothed before fitting Eq. (4a).

## 3 Results

We report some selected results of fits of the four models to the data for demonstrative purposes, that is, we select biomass time series of specific genotypes in specific trials to demonstrate the fitting of our minimalistic crop growth models. In all Figures, black indicates the noise-free APSIM biomass data, and green indicates the fit of our minimalistic crop growth model to the biomass data.

**Figure 1:**
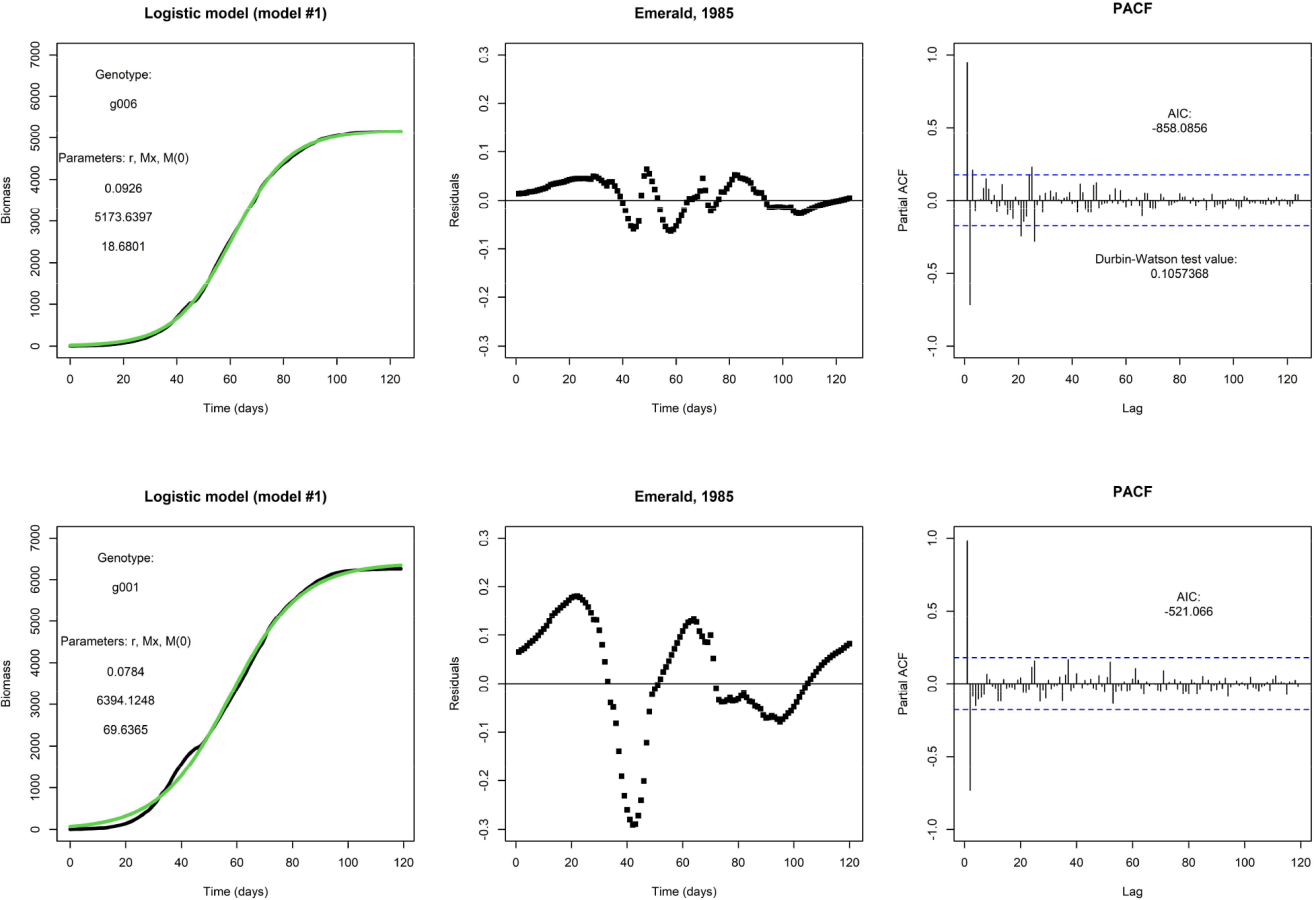
Top row: An example fit of the logistic model, with Emerald, 1985, g006. Fitted parameter values are displayed. Visually, the fit (in green) matches nicely with the daily biomass series (in black). Bottom row: An example of the same model and same environment, but genotype g001. The fit is visually less satisfactory than the fit for genotype g006.

**Figure 1** shows two examples of a fit of the logistic model (model #1). The top row shows the fit for one genotype (g006), and this particular fit is visually satisfactory. It will be no surprise that the weighted residuals (see top middle panel) are judged as not being i.d.d. according to the Durbin-Watson statistic, and they are clearly autocorrelated, as can be seen from the PACF (see top right panel). However, the autocorrelation is not an obvious reason to reject this model, and the fit suggests that a logistic model would be adequate for prediction purposes. At this point, one may argue the need for the inclusion of any additional explanatory factor. However, repeating an earlier point, the logistic model is fixed by the initial condition and does not allow for modifications in the growth rate by external inputs during the growing season. This would present a more pressing reason for any rejection of the model. In the bottom row, we fit the same model to the same environment but for a different genotype (g001); note that this genotype also takes fewer days to get to end-of-season. This fit is less satisfactory and the residuals plotted in time make larger excursions.

**Figure 2:**
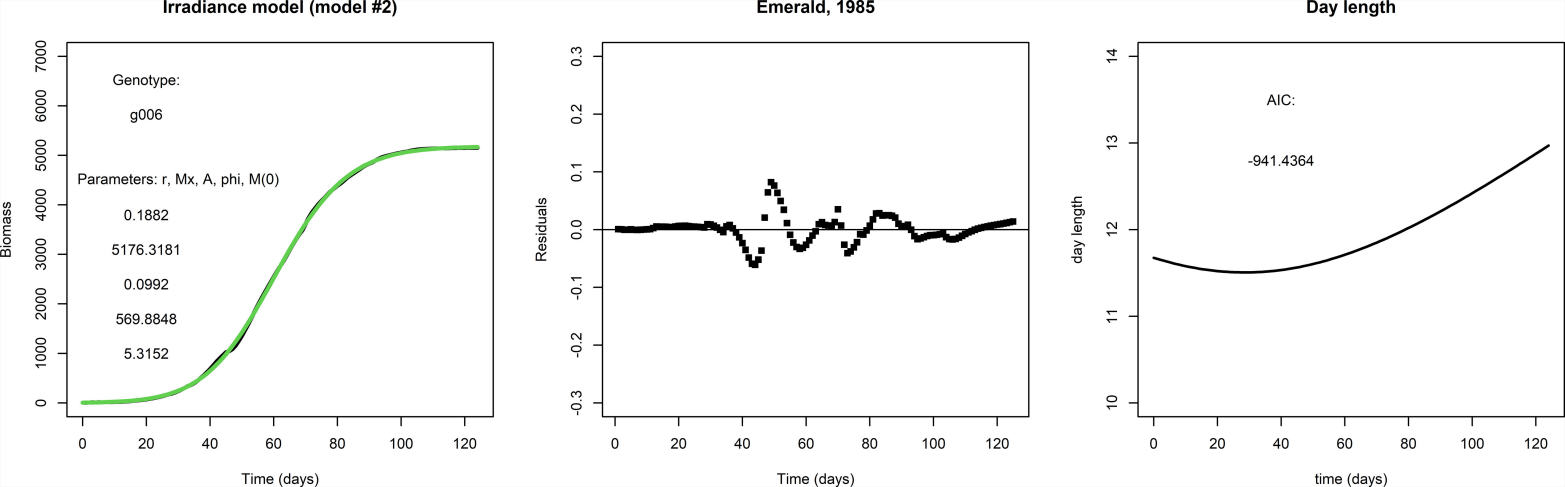
Left panel: A fit with the irradiance model to the same data set used to fit the logistic model in **Figure 1**, lower row. Middle panel: The residuals and AIC suggest that the irradiance model is a considerable improvement over the logistic model in this particular case. Right panel: Day length, taken from the APSIM model.

**Figure 2** gives an example of how the fit to the data may be improved by the irradiance model (model #2). The fit for g001 with the irradiance model is considerably better than that with the logistic model: the AIC score is much lower, the residuals are smaller and even close to zero in the early and late parts of the growing season, and the initial condition *M*(0) is much smaller. This suggests that the addition of two parameters for the seasonality is an improvement, and also suggests that the inclusion of day length as approximation of irradiance is worthwhile.

**Figure 3:**
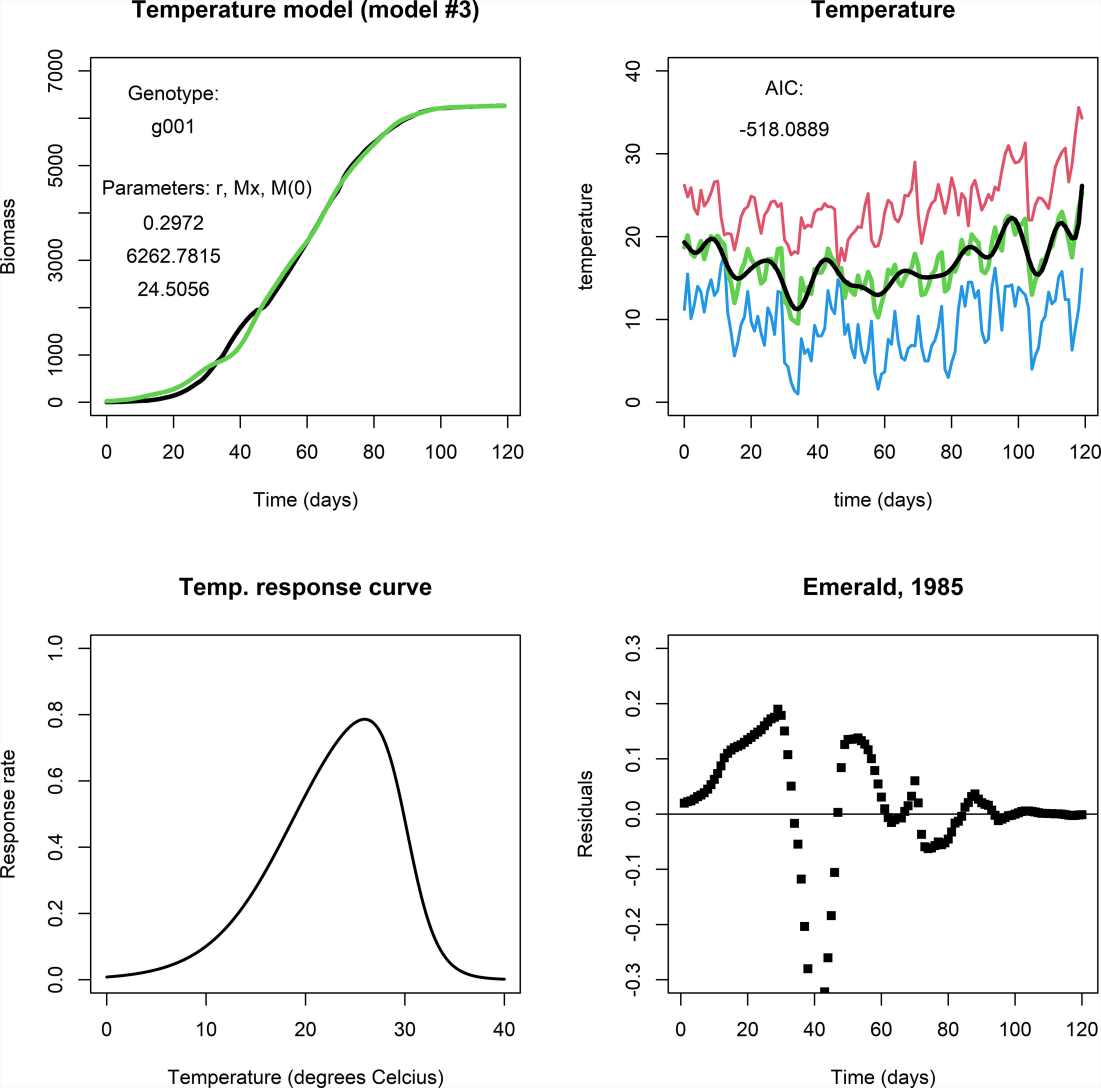
Upper left panel: A fit with the temperature model for Emerald, 1985, g001. Upper right panel: Red indicates maximum daily temperature, blue minimum, green the average temperature, and black the smoothened curve. Lower left panel: The temperature response curve, which was fixed in all simulations with the temperature model. Lower right panel: The fit mostly follows the biomass data during the growing season, but in particular in the period between day 25 through 50 the residuals are far off. This may be an indication that the temperature response curve should be adjusted, or that temperature is not the main factor that affects growth at this stage of the growing season.].

The temperature model exhibited more dynamic changes to within-season conditions for growth because it involves an exogenous variable (temperature). For comparison, **Figure 3** shows a fit for genotype g001 in the same environment as the previous two models (upper left panel). It captures modifications to biomass growth rate that are relatively small and that took place on the scale of a couple of days (see **Figure 3**, upper right panel), producing realistic end-of-season biomass predictions. **Figure 3**, lower left panel shows the temperature response curve using Eq. (3b) and the parameter values given in **Table 3**. For many of our genotype by environment combinations, this model did not improve the fit in comparison to the fits by the logistic model or irradiance model, e.g., consider the plot of the residuals (**Figure 3**, lower right panel). Note, that the parameters in Eq. (3b) were kept constant across genotypes and environments, and that different results may be obtained when the temperature response is adjusted. Also, in most of our simulated data, temperatures varied mildly within the (sub) optimal range for wheat growth, so it is perhaps not surprising that the temperature model did not translate to a considerable improvement in describing biomass development compared to the fits of the logistic model and the irradiance model.

**Figure 4:**
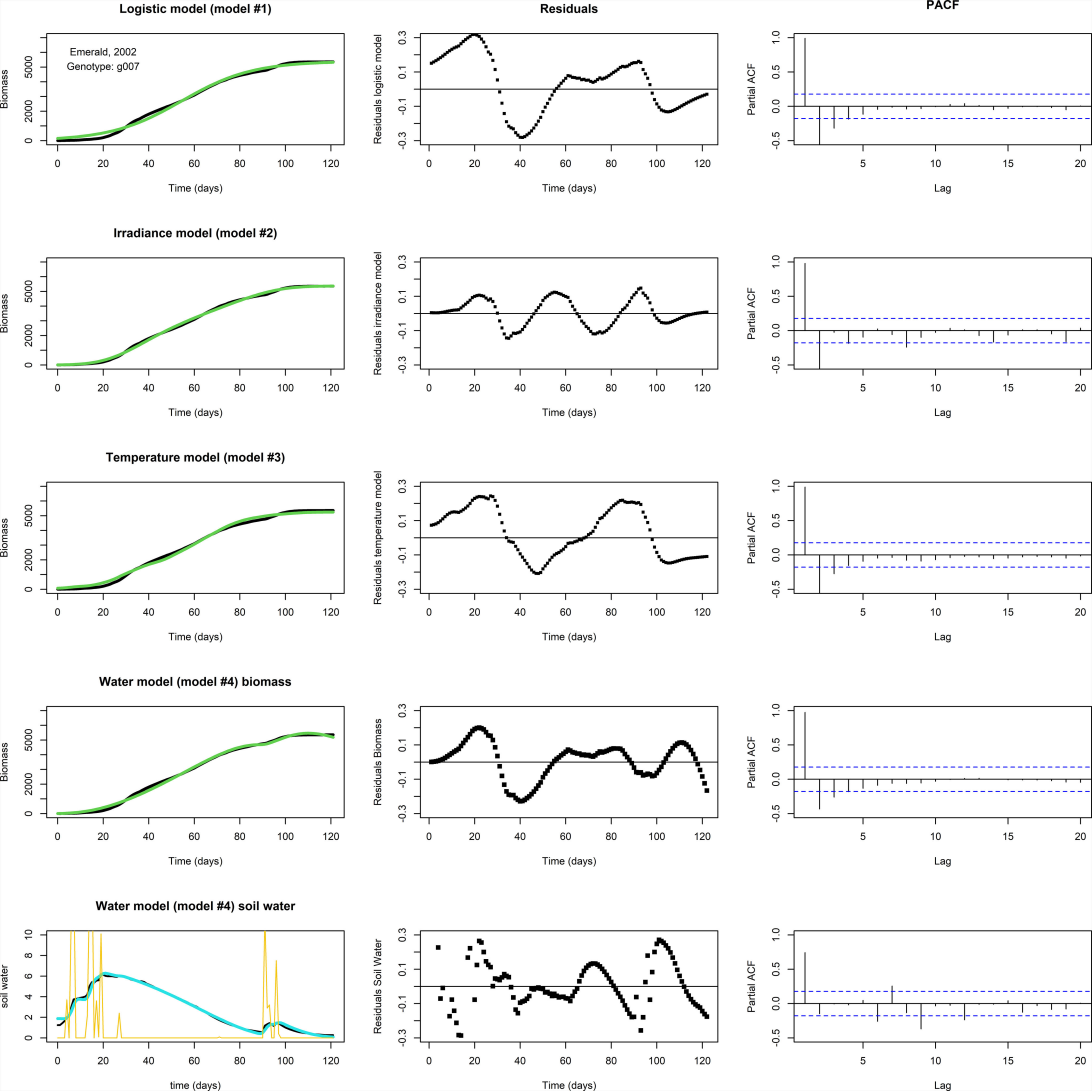
Fits to data set Emerald, 2002, g007. The biomass data (in black) shows a ‘bump’ around days 90-95. Left panels: Optimized fit of the model to the data. Middle panels: Residuals of the optimized fit. Right panels: PACF. Top row: A fit with the logistic model. Second row: The irradiance model. Third row: The temperature model. None of the models show a satisfactory fit. Fourth row: A fit of biomass with the water model, using Nelder-Mead, with resulting parameter values *p*=1.061, *K*=3.618·10^2^, *q*=5.521·10^–2^, *c*=7.824·10^–2^, *n*=1.095, *v*=5.318·10^–1^, *R*=1.320·10^–3^, *g*=1.108, *m*=1.150·10^–2^, *W*(0)=1.880, and *M*(0)=5.584·10^–3^. Lower row: The fit of the soil water (in blue). Precipitation is plotted in yellow. Note the decrease in biomass near the end of the growing season in the water model fit, which is likely the result of an overestimated maintenance, the main term in the model that can lead to biomass reduction.

None of the three models above, logistic, irradiance, or temperature, produced adequate fits for the biomass longitudinal data of genotype by environment combinations in which a ‘bump’ occurred near the end of the growing season (**Figure 4**, the top three rows, for an example involving g007, growing in Emerald, 2002). In this subset of genotype by environment combinations, there was a considerable biomass increase before the usual levelling-off at the end of the growing season, which seemed to coincide with changes in water availability. **Figure 4**, fourth row gives the biomass fit of the water model (Eq. 4b), including the (PACF of the) residuals of the biomass, while the bottom row gives the fit of the soil water (Eq. 4a), including residuals. In this set, the ‘bump’ started around day 92. The temperature remained approximately stationary for several days around day 92, while soil water levels were increased by precipitation around days 90-95 (**Figure 4**, lower left panel), suggesting that a depletion of soil water was the main driver responsible for the levelling off of the growth rate, at least towards the second half of the growing season. The water model qualitatively shows the same development in biomass and soil water as the data, though the residuals indicate that the fit is not perfect. This could be the result of an overestimated maintenance, the main term for biomass loss in the model, which was assumed to be scaling linearly with biomass. Also, growth in the first half of the growing season may have been limited not by soil water but by another factor.

## 4 Discussion

In this paper we considered parsimonious crop growth models consisting of differential equations with few parameters and variables that couple the rate of change in biomass to its own state. The aim was to present a modelling framework of limited complexity for genotype-dependent trait prediction involving essential GxExM interactions and that can respond to changes during the growing season as measured by proximal and remote sensing. The four presented models each had a different limiting environmental factor: a generic limitation described by logistic growth, irradiance via day length, temperature response, or soil water availability, respectively. The models were fitted to *in silico* longitudinal data of 199 genotypes generated with APSIM-Wheat to demonstrate their performance. In many cases the logistic model gave already quite an adequate fit, but for many genotype by environment combinations there was at least one alternative model that matched the data convincingly better. This implies that for such genotype by environment combinations a specifically identified environmental limitation was dominant.

Depending on the specific combination of genotype and environment, where an environment refers to a trial simulated for a combination of year and location, one model may fit better than another. The calibration procedure may be limiting as well. In those situations in which we judged the logistic model to be adequate, we found that fitting this model by FME – deSolve was unproblematic, and often a single attempt at fitting this model sufficed. Fitting of the temperature model was more problematic and required the temperature response parameters in Eq. (3b) to be fixed, as leaving one or more of these parameters free in fitting in many cases led to temperature response curves that were unrealistic, as assessed by visual inspection. The water model proved most challenging in fitting and usually required several fitting attempts. The first term in Eq. (4a) involving the infiltration of precipitation in the soil turned out to be an essential part, as replacing the term with a linear response function of precipitation resulted in suspect and unrealistic fits (not shown). Note, that when we are fitting each combination of genotype and environment separately – like we did in this paper – then there are nine parameters and two initial conditions to fit. This makes it likely there are multiple local fitting optima, which may lead to spurious results. The use of alternative fitting approaches may produce better results. In a hierarchical approach to fitting we can divide the parameters into two groups that can be fitted separately: one group of parameters that vary across environments – and that hence should be approximately constant across genotypes grown in the same environment – and one group of parameters that mainly vary across genotypes.

While there is a decades-long history of developing crop simulation platforms (Jin et al., 2018), it remains challenging to balance a broad application range with model parsimony. The limited complexity of the proposed models in this paper hampers a more general application. In these models only one limitation is included. In reality, it is likely that two or more limitations occur during a growing season, and the particular limiting factor may change during the growing season: important limiting factors like irradiance, atmospheric carbon dioxide, soil water, and nutrients may operate at different moments and scales. Also, multiple limitations may occur at the same time. As an example of this phenomenon, Zhao et al. (2022) attributed considerable losses of winter wheat to the co-occurring extreme events of high temperatures, dryness and hard winds compared to events in which a single limiting factor occurred. Additionally, what parsimony offers in terms of elegance and statistical ease may come at the cost of fewer points of entry for discriminating among genotypes. In particular, the models proposed in this paper ignore phenology. Yet, the examples shown in this paper suggest one can already explain much of the crop growth in some situations with low-complexity models that ignore phenology.

Some dynamic crop models have been proposed that include essential processes and that try to balance limited model complexity with a broad application range. For example, the SIMPLE model consists of 13 parameters, involving cumulative temperature for phenological development and a multiplicative function including daily temperature, heat stress, drought stress, atmospheric carbon dioxide concentration, radiation use efficiency, and irradiance interception, and has been parameterized for 14 different crop species (Zhao et al., 2019).

A second step in modelling involves the introduction of functions that can smoothly and dynamically describe multiple resource limitations that occur during the growing season, i.e., that involve multiple co-limitations at different points in time and space. Arguably the best known multiple limitation description is Liebig’s law of the minimum, where it is assumed there is only a single resource limitation at any given time, while any switch to another limitation is instantaneous (Cossani and Sadras, 2018). Functions that allow for a smoother transition to another limiting factor or that allow for multiple co-limiting factors have also been proposed in ecological models (Dutta et al., 2014). One example is the Synthesizing Unit (SU; Kooijman, 2010), which is a functional response comparable to the Michaelis-Menten (MM) functional response. Like the MM functional response, the SU is derived from law of mass action kinetics and includes multiple factors that are unique or mutually exchangeable in a smooth and continuous model formulation (Dutta et al., 2014). The SU has, for instance, been used to capture the co-limitation of light and nitrogen in the growth of heather shrubs and wavy hair-grass (Van Voorn et al., 2016). Another example is the description of the co-limitation of light, carbon dioxide, nitrogen, and phosphorus in the growth of cyanobacteria that were grown in a set of experiments across which these environmental factors were simultaneously varied in different combinations of higher and lower concentrations (Grossowicz et al., 2017).

With the inclusion of multiple limitations for the description of GxExM interactions comes the need for stronger data requirements for model fitting and reduction of uncertainties. In particular, the water model consisted of several parameters, initial conditions, and one explicit exogeneous input (precipitation). This model turned out to be more difficult to fit, even to the noise free data we had available. Furthermore, despite increasing data availability, proximal sensing data are still relatively rare and expensive. The fitting of the water model was further complicated by a conceptual mismatch between the soil water variable in our crop growth model (where it is an independent state variable interacting with the crop biomass) and the soil water supply variable in the APSIM simulated data (where it is calculated based on precipitation and available soil water following a submodule). Also, uncertainties remain that negatively affect forecasting. For instance, imprecision in soil, canopy state, and meteorological data propagate to uncertainty in model predictions (Hansen & Jones, 2000; Grassini et al., 2015), while various random events occur during the growing season, such as changes in irradiance due to clouds, irregular precipitation, or temperature changes.

In the current paper, we fitted dynamic models to longitudinal biomass data consisting of time series data corresponding to a single genotype in a single environment (year by location). We used an approach especially developed for fitting differential equations, focussing on the estimation of rates of change and initial conditions (Soetaert and Petzoldt, 2010; Soetaert et al., 2010). We concluded that the four minimalistic models may be useful to describe biomass dynamics in wheat. However, our ultimate aim goes far beyond the fitting of dynamical models for individual genotypes in individual environments. In the end, the goal is to develop a modelling framework for describing dynamical phenotypic behaviour of genotypes as functions of genetic (QTLs, SNPs in genomic prediction), management and environmental inputs. Such a modelling framework should allow us to predict yield and other traits for existing and new genotypes across a wide range of management and environmental conditions, and produce GxExM interactions in an emergent way. Leading contributions to the creation of such a modelling and prediction framework include papers by Technow et al. (2015), Cooper et al. (2016), and Messina et al. (2018). The starting point for these papers were existing crop growth models in which a small number of crop growth parameters were made genotype-dependent by regressing them on marker profiles inside a hierarchical Bayesian model. In the current paper, we intend to simplify the extensive dynamical crop growth model that was used in the papers mentioned above and reduce it to a small number of differential equations with a limited number of parameters. The challenge that we need to address is to identify a suitable dynamic model for each combination of genotype and environment and estimate genotype-dependent dynamic parameters, with little or no genotype by environment interaction, that can be made functions of marker profiles. Our hope is firstly that we will be able to model the dynamic behaviour of longitudinal traits as produced by especially proximal sensing devices by simple differential equations in a data driven way. Secondly, because the proposed differential equations have a limited number of parameters, we should be able to estimate genetic variation in the rate parameters. Of course, when explicit solutions exist to the differential equation system, a statistical approach to our problem could be found in the application of non-linear mixed models (Pinheiro and Bates, 2000). Without such explicit solutions, Bayesian hierarchical approaches may offer the best perspectives (Poudel et al., 2022), which is the route we will explore in a follow-up paper.

With dynamic models that incorporate data assimilation, the forecasting solution by the model may be adapted during the growing season. Remote and proximal sensing methodologies can provide actual state estimations at different scales, that can be used to update the state estimations by the crop model and thus steer forecasting by the model. Different assimilation approaches, including Particle Filter and Hierarchical Bayesian Method, have been proposed (Jin et al., 2018). For example, an Ensemble Kalman filter (EnKF) was used to assimilate sensor data of soil moisture to correct errors in the water balance of the WOFOST crop model (De Wit & Van Diepen, 2007). A similar approach was followed for constraining uncertainty in an upstream process in the model APSIM, with the goal of reducing uncertainty in the downstream processes (Kivi et al., 2022). Data assimilation for prediction updating also is a method for including exogenous input changes in ‘static’ models, such as the logistic growth.

Crop modelling has a critical role in forecasting crop growth of existing and new genotypes in existing and new environments. Future efforts for crop growth prediction should aim at a strategy of balancing crop model complexity: on the one hand, these models need to be process-based and describe multiple essential GxExM interactions to predict crop growth across a large genetic spectrum and multiple environments, while on the other hand they have to remain parsimonious to constrict issues around parameter fitting and uncertainty. This requires large-scale longitudinal data, that may become available with the increased remote and proximal sensing. More advanced fitting approaches should be tailored to support this strategy, while data assimilation can be used to reduce prediction uncertainty.

## Data availability statement

The original contributions presented in the study are included in the article/Supplementary Material. Further inquiries can be directed to the corresponding author.

## Author contributions

Modelling GxExM was conceived between Corteva Agriscience™ and Wageningen University and Research – Biometris and led by CM and FV, respectively. Conceptualization and development was done by all authors. APSIM-Wheat simulation data were created by DB. Model fitting and first version manuscript writing and preparation was done by GV. Intermediate editing of manuscript was done by all authors. FV and GV did the final manuscript editing. All authors contributed to the article and approved the submitted version.
